# Sexual symptoms in relation to curative pelvic radiotherapy in gynecological cancer patients

**DOI:** 10.1007/s11764-025-01916-z

**Published:** 2025-10-24

**Authors:** Annica Knutsen, Rasmus Mikiver, Kristina Redelius, Srinivas Uppugunduri

**Affiliations:** 1 Department of Biomedical and Clinical Sciences, Linköping University, Linköping, Sweden; 2 Department of Oncology, County Council of Östergötland, Linköping University, S-58185 Linköping, Sweden; 3 Regional Cancer Centre Southeast Sweden, Linköping, Sweden

**Keywords:** Cervical cancer, Uterine tumors, Radiotherapy, Sexual symptoms, Side effects

## Abstract

**Purpose:**

Pelvic radiotherapy (RT) causes tissue injury which could lead to vaginal problems including dryness, shortening, and tightening of the vagina, causing discomfort, and affect sexual activity. In this longitudinal, prospective study, the association between pelvic RT and sexual function were studied in primary cervical cancer and in primary uterine tumor patients.

**Methods:**

This prospective longitudinal cohort study comprised patients with primary cervical cancer and primary uterine tumors with pelvic radiotherapy (RT). Seventy-nine cervical cancer and 43 uterine tumor patients were included from May 2014 to February 2019. Patients completed questionnaires for global health status and sexual functioning before RT start and at 3 and 12 months after RT.

**Results:**

In primary cervical cancer patients, the sexual activity was significantly higher in patients with pelvic RT alone compared to patients with pelvic RT + cervical brachytherapy/boost at 3 months (*P* = 0.007, 34.6% vs. 73.3%) and 12 months after RT (*P* = 0.054, 45.7% vs. 76.9%). No significant relationship was found in primary uterine tumor patients. At 12 months after RT, 52.5% of the cervix and 42.3% of the primary uterine tumor patients were sexually active. Of the cervical cancer patients, 81.8% reported symptoms as dry vagina 12 months after RT. Local estrogens did not improve the sexual activity in cervical cancer or uterine tumor patients after RT.

**Conclusion:**

In cervical cancer patients, the sexual activity was significantly higher with pelvic RT alone compared to pelvic RT + cervical brachytherapy/boost. Better medical interventions are needed to reduce the sexual symptoms.

**Implications for Cancer Survivors:**

Reduced sexual activity in gynecological cancer survivors with curative pelvic RT. For the future, improved medical interventions are needed to decrease the sexual symptoms.

## Introduction

High doses of curative pelvic radiotherapy (RT) are administered to cervical cancer patients in stage IB-IV [1] and also to reduce postoperative locoregional relapses in primary endometrial cancer patients [2, 3]. During recent years, curative radio-chemotherapy for cervical cancer patients and postoperative radiotherapy for patients with primary uterine tumor has proven to be very effective, and today, more than 80% of the patients survive their disease.

Despite its significant benefit on locoregional relapses and survival, pelvic RT is associated with severe tissue injury, which could give rise to many side effects such as gastrointestinal and urologic symptoms, pelvic pain, lymphedema, and sexual symptoms [4–6].

The sexual symptoms consist mainly of a loss in physical function including vaginal atrophy, shortening, tightening and pain, which could make it very difficult to have sexual intercourse [7, 8]. It could also affect the psychological aspects of sexuality like changes in the perception of body image, which could further lead to decreased sexual interest, sexual enjoyment and activity [8–10].

The frequency of sexual symptoms in patients with primary cervical and uterine tumor has been studied by others [4, 5, 7, 9, 11, 12] but no recent study has analyzed the association between the frequency of sexual symptoms and different types of RT in primary cervical cancer patients. The relationship between pelvic RT and the sexual side effects was assessed in a previous study involving primary cervical cancer patients treated with radio-chemotherapy. In this study, all patients (100%) had RT with additional brachytherapy/boost to the cervix and no comparison between RT alone and RT with brachytherapy/boost were performed [11].

The association between the frequency of sexual symptoms and type of pelvic RT in primary uterine tumor patients has been investigated in a single study [9]. In this previous study, the EORTC-CX-24 questionnaire, specific for cervical cancer patients, was used to assess sexual symptoms, whereas we have used the EORTC-EN-24 questionnaire specific for primary endometrial cancer patients. In this previous study by others, no significant differences in frequency of sexual symptoms between patients with pelvic RT alone or pelvic RT + vaginal brachytherapy was found [9].

The aim of this prospective study was to evaluate the incidence and time course of the side effect as sexual symptoms in relation to pelvic RT treatment in patients with primary cervical cancer and uterine tumors and to further study sexual symptoms in relation to type of pelvic RT.

## Material and methods

### Patients

This study included 79 patients with primary cervical cancer and 43 patients with primary uterine tumors. All patients received curative pelvic RT from May 2014 to February 2019. The patients were treated at the Department of gynecological oncology, University Hospital, Linköping, Sweden. All patients with primary cervical cancer patients who received either primary or postoperative curative pelvic radio-chemotherapy and all uterine tumor patients with adjuvant postoperative pelvic RT were asked to participate in the study. All patients had treatment according to the national guidelines. The mean age for the cervical cancer patients was 52 years (range 28–84 years) and 62 years (range 39–76 years) for the uterine tumor patients. Patients with dementia, or those unable to read and speak Swedish, and patients not capable of receiving standard adjuvant treatment (co-morbidities and/or poor performance status) were excluded from the study.

The study was in accordance with the Declaration of Helsinki and approved by the regional ethical committee in Linköping, Sweden (Reference number: 2018/363–31, 2019/013–33). All patients had signed a written informed consent prior to the study start.

### Data

Data such as age (at the time for diagnosis), differentiation grade, stage, time of diagnosis, date of surgery, type of surgery, type of curative/postoperative radiotherapy treatment, local and systemic estrogen treatment, performance status, were obtained from the patient’s oncological and surgical files (Tables 1 and 2).

**Table 1:** Patient characteristics of the 79 primary cervical cancer patients

Variables	
Cervix *N* = 79	*n* (%)
Age (mean)	52 years (range 28–84)
Stage	
IB1-IB2	25 (31.6)
IIA1-IIB	35 (44.3)
IIIA-IIIB	15 (19.0)
IVA	4 (5.1)
Histology	
Squamous cell carcinoma	57 (72.2)
Adenocarcinoma	17 (21.5)
Adenoskvamous carcinoma	2 (2.5)
Others	3 (3.8)
Type of surgery	
No surgery	56 (70.9)
TAH + SOEB + pelvic lymphadenectomy	18 (22.8)
TAH + SOEB	5 (6.3)
Chemotherapy	
No	8 (10.1)
Yes	71 (89.9)
Type of chemoradiotherapy	
2 Gy × 25 pelvis	13 (16.5)
1.8/2 Gy × 25 pelvis CBT* 6Gy × 4 and/or boost 2Gy × 7–8	66 (83.5)
Performance status (WHO)	
0	67 (84.8)
1	10 (12.7)
2	2 (2.5)
Other diseases	
No	47 (59.5)
Yes	32 (40.5)
Local estrogen treatment	
No	59 (74.7)
Yes	20 (25.3)
Systemic estrogen treatment	
No	54 (68.4)
Yes	25 (31.6)

*CBT, cervical brachytherapy

**Table 2:** Patient characteristics of the 43 primary uterine tumor patients

Variables	
Uterine *N* = 43	*n* (%)
Age (mean)	62 years (range 39–76)
Stage	
I	4 (9.3)
II	10 (23.3)
III	28 (65.1)
IV	1 (2.3)
Histology	
Endometrioid grade 1–2	15 (34.9)
Sarcoma (leiomyosarcoma + low-grade endometriostroma cell sarcoma)	4 (9.3)
Endometroid grade 3 + clear cell + serous + carcinosarcoma	24 (55.8)
Adjuvant chemotherapy	
No	9 (20.9)
Yes	34 (79.1)
Type of adjuvant radiotherapy	
45–46 Gy pelvic ± paraaortic EBRT	14 (32.6)
45–46 Gy pelvic ± paraaortic EBRT + VBT* 2–4 Gy × 4/Boost 2 Gy × 6–7	29 (67.4)
Performance status (WHO)	
0	40 (93.0)
1	2 (4.7)
2	1 (2.3)
Other diseases	
No	19 (44.2)
Yes	24 (45.8)
Local estrogen treatment	
No	33 (76.7)
Yes	10 (23.3)
Systemic estrogen treatment	
No	42 (97.7)
Yes	1 (2.3)

*VBT, vaginal brachytherapy

### Quality of life assessment

For patients with primary cervical cancer, two questionnaires the EORTC QLQ-C30 and the EORTC QLQ-CX-24 were handed out. For patients with primary uterine tumors, the EORTC QLQ-C30 and the EORTC QLQ-EN24 was used. The questionnaires were handed out at baseline (the first consultation with a radiation oncologist 3–4 weeks before start of RT) and further sent to the patient’s home address at 3 and 12 months after finishing RT treatment. The EORTC QLQ-C30 is a multidimensional quality of life questionnaire used in clinical trials for cancer patients [13]. The EORTC QLQ-CX-24 and EORTC QLQ-EN24 contains more specific questions concerning the side effects observed in cervical and endometrial cancer patients [14, 15]. The response scale varied between 1 to 4 and was used for each item. All subscales reported were converted to 0–100 scales [16].

### Statistics

Non-parametric Wilcoxon signed rank test was used to study the scores of sexual symptoms between paired samples before the start of RT, at 3 months and 12 months after RT. The Chi-square (X^2^) test and the Fischer´s exact test was used to analyze sexual activity in relation to different RT treatments and local/systemic estrogen treatment. Matched cases were used for all comparisons. The software program STATISTICA (version 13.5) and the SPSS: IBM Corp. Released 2020. IBM SPSS Statistics for Windows, Version 27.0. Armonk, NY: IBM Corp R Statistical Software (v4.0.3; R Core Team 2020; https://www.r-project.org/) were used for all the statistical analyses. A two-sided test was used and a *p*-value of *P* < 0.05 was considered statistically significant.

## Results

### Study population

In total 103 cervical cancer patients and 52 patients with primary uterine tumors were invited to complete the EORTC QLQ questionnaires at the start of RT. Seventy-nine (76.7%) of the cervical cancer patients and 43 (82.7%) of the uterine tumor patients answered the questionnaire at least once. Of the 79 cervical cancer patients who answered the questionnaires, 63 (79.7%) answered the forms before RT, 71 (89.9%) at 3 months and 51 (64.6%) 12 months after RT. Of the 43 uterine tumor patients, 40 (93.0%) answered the questionnaires before RT, 38 (88.4%) at 3 months and 32 (74.4%) 12 months after finishing RT. Of the 103 cervical cancer patients 24 (23.3%), and of the 52 uterine tumor patients, 8 (15.4%) deceased during enrollment.

### Treatment

The external beam radiotherapy (EBRT) was given with 1.8/2 Gy × 25 to the pelvis of patients with primary cervical cancer. Sixty-six (83.7%) of the patients received additional cervical brachytherapy (CBT) (6 Gy × 4) and/or pelvic boost (2 Gy × 7–8). For patients with primary uterine tumors the EBRT was given with 46–50 Gy in 23–25 fractions to the pelvis or 45 Gy in 25 fractions given to the pelvis and para-aortic lymph nodes. Twenty-nine (67.4%) out of 43 the uterine tumor patients had additional brachytherapy (2-4 Gy × 4)/boost (2 Gy × 6–7) to the vagina/parametrium (Tables 1 and 2). All treatments were administered with intensity modulated radiotherapy (IMRT). Seventy-one (89.9%) out of the 79 cervical cancer patients received concomitant chemotherapy and 34 (79.1%) of the 43 uterine tumor patients had adjuvant chemotherapy before RT (Tables 1 and 2).

### Symptom score

**Fig. 1:**
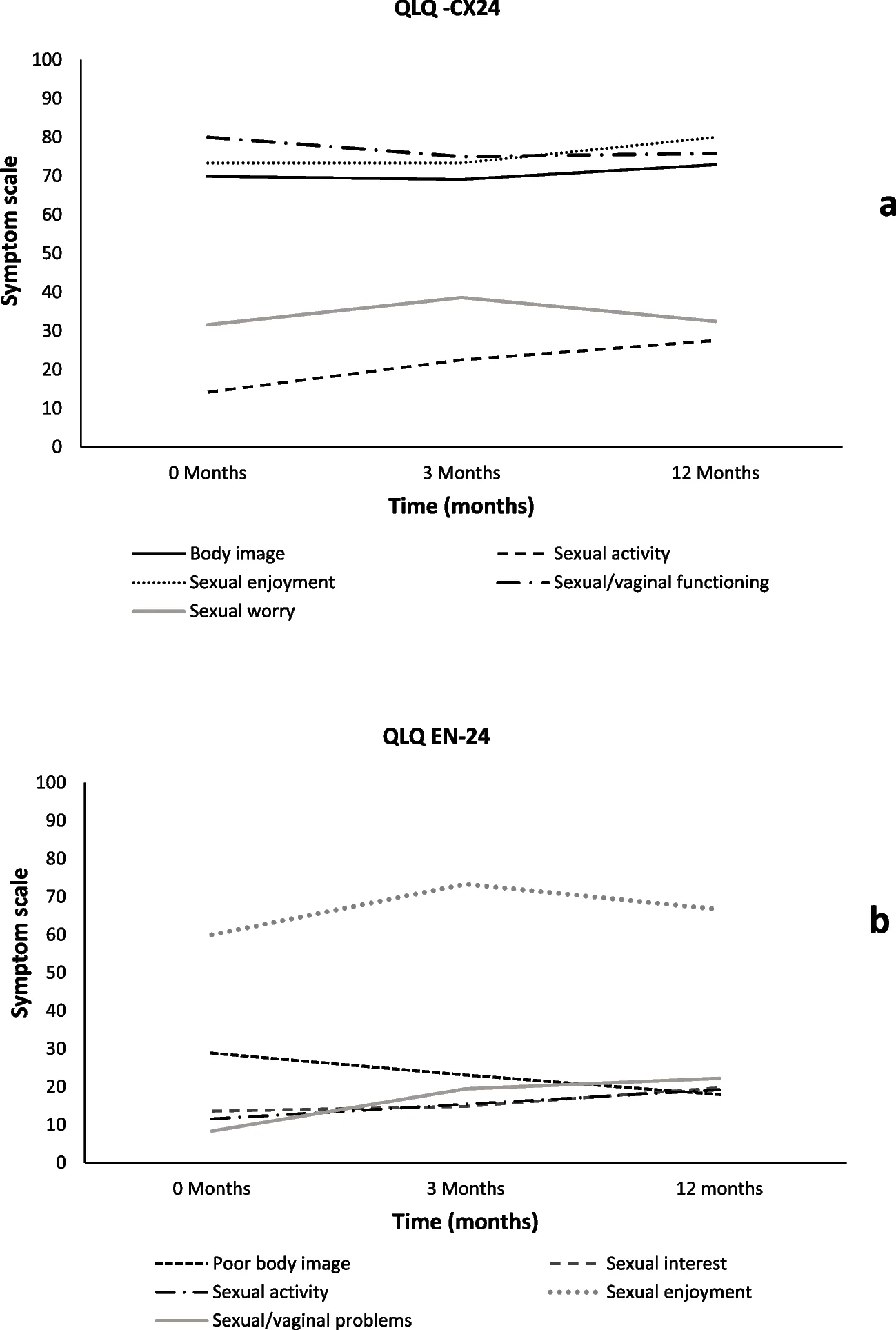
In primary cervical cancer patients (**a**). Symptom scoring for body image, sexual enjoyment, sexual activity, sexual/vaginal functioning before RT, and 3 months and 12 months after RT. In primary uterine tumor patients (**b**). Symptom scoring for poor body image, sexual activity, sexual/vaginal problems, sexual interest and sexual enjoyment before RT, and 3 months and 12 months after RT

The symptom scores were studied by using the EORTC QLQ-CX24 for patients with cervical cancer and EORTC QLQ-EN24 for patients with uterine tumors with RT. In patients with primary cervical cancer the sexual activity significantly increased 12 months after RT compared to before RT (*P* = 0.002, Fig. 1A, Table 3). The sexual enjoyment also significantly improved 3 months after RT compared to values at RT start (*P* = 0.020). Further, we found a significant increase in the sexual interest and trend towards increased sexual activity in patients with primary uterine tumors 12 months after RT compared to values at RT start (*P* = 0.025, *P* = 0.058, Fig. 1B, Table 3).

**Table 3:** Data of the global health status, functioning, and symptom score from the EORTC QLQ-C30, QLQ-CX24, and QLQ-EN24 questionnaires before RT and 3 and 12 months after RT in patients with primary cervical and uterine tumors

Questionnaire, functioning, and symptoms	Before RT vs. 3 months after RT				3 months after RT vs. 12 months after RT				Before RT vs. 12 months after RT			
	Before RT	3 months after RT	n*	*p*-value	3 months after RT	12 months after RT	n*	*p*-value	Before RT	12 months after RT	n*	*p*-value
EORTC QLQ-C30 functioning scale (CX24)												
Global health status	61.1	59.2	57	0.485	64.3	69.0	49	0.043	59.9	68.5	42	0.008
Cognitive function	76.3	78.9	57	0.371	82.0	84.7	49	0.336	77.0	83.7	42	0.045
Emotional function	63.3	68.0	57	0.018	70.2	78.9	49	0.002	62.0	78.3	42	< 0.001
EORTC QLQ-CX24 symptom scale												
Body image	68.3	66.1	56	0.600	68.0	72.8	49	0.114	70.4	72.8	42	0.499
Sexual activity	11.5	18.2	55	0.022	21.7	27.5	46	0.090	13.8	26.8	41	0.002
Sexual worry	25.8	34.6	53	0.099	37.8	29.6	45	0.025	30.8	33.3	40	0.659
Menopausal symptoms	37.5	44.6	56	0.087	42.9	33.3	49	0.056	38.9	31.0	42	0.184
Sexual enjoyment	66.7	61.5	13	0.581	56.9	70.6	17	0.020	66.7	82.1	13	0.161
Sexual/vaginal functioning	84.0	80.8	13	0.383	76.5	77.9	17	0.501	78.8	76.3	13	0.692
EORTC QLQ-C30 functioning scale (EN24)												
Global health status/QoL	65.3	66.9	36	0.518	68.8	72.6	28	0.430	63.9	70.7	27	0.145
Cognitive function	87.1	85.2	35	0.526	87.8	89.4	30	0.470	90.5	88.7	28	0.587
Emotional function	76.0	76.7	35	0.776	78.9	85.6	30	0.014	74.4	84.5	28	0.018
EORTC QLQ-EN24 symptom scale												
Poor body image	27.0	24.0	34	0.424	22.6	16.7	28	0.166	28.0	18.5	28	0.129
Sexual interest	13.9	13.9	36	1.000	14.3	19.0	28	0.046	13.6	19.8	27	0.025
Sexual activity	11.4	14.3	35	0.257	14.3	17.9	28	0.180	11.5	19.2	26	0.058
Sexual enjoyment	50.0	62.5	8	0.083	62.5	58.3	8	0.317	60.0	66.7	5	0.564
Sexual/vaginal problems	14.3	22.2	7	0.197	16.7	22.2	8	0.206	8.3	22.2	4	0.197

*All analyses were based on matched cases

### Number of patients with symptoms

Next, we studied the presence of sexual symptoms in the group of sexually active primary cervical cancer patients with RT (*n* = 40). The number of patients who were sexually active increased slowly from RT start to 12 months after RT. The sexual activity was 32.5% at RT start and increased to 47.5% and further to 52.5% at 3 and 12 months after RT. A high percentage of patients (73.7% out of 38 patients) reported concerns that it would hurt during sexual intercourse at 3 months after RT. Also, 63.4% out of 41 patients, felt dissatisfied with their body 12 months after RT (Table 4). In the cervical cancer patients that were sexually active and had answered the questionnaires (*N* = 11), almost half of the patients (45.5%) exhibited symptoms as dry vagina during sexual intercourse at RT start, which increased to 81.8% 12 months after RT (Table 4).

**Table 4:** The percentage of patients with sexual symptoms before RT and 3 and 12 months after RT in primary cervical and uterine tumors

Symptoms	^n**^	Before RT	3 months after RT	12 months after RT
		%^*^	%^*^	%^*^
ENGOT-CX24 questionnaire				
Sexual symptoms				
Have you had irritation or soreness in your vagina or vulva?	40	47.5	55.0	52.5
Have you had hot flushes and/or sweats?	41	61.0	80.5	56.1
Have you felt physically less attractive as a result of your disease or treatment?	41	58.5	58.5	53.7
Have you felt less feminine as a result of your disease or treatment?	41	43.9	39.0	41.5
Have you felt dissatisfied with your body?	41	58.5	58.5	63.4
Have you worried that sex would be painful?	38	47.4	73.7	55.3
Have you been sexually active?	40	32.5	47.5	52.5
Answers only from those who have been sexually active the latest 4 weeks				
Has your vagina felt dry during sexual activity?	11	45.5	81.8	81.8
Has your vagina felt short?	10	50.0	60.0	60.0
Has your vagina felt tight?	10	50.0	50.0	30.0
Have you had pain during sexual intercourse or other sexual activity?	10	30.0	70.0	50.0
Was sexual activity enjoyable for you?	10	100.0	100.0	100.0
ENGOT-EN24 questionnaire				
Sexual symptoms				
Have you felt physically less attractive as a result of your disease or treatment?	26	57.7	53.8	38.5
Have you felt less feminine as a result of your disease or treatment?	26	50.0	42.3	38.5
To what extent were you interested in sex?	27	29.6	40.7	40.7
To what extent were you sexually active?	26	30.8	42.3	42.3
Answers only from those who have been sexually active the latest 4 weeks				
Has your vagina felt dry during sexual activity?	4	50.0	75.0	50.0
Has your vagina felt short and/or tight?	5	40.0	20.0	60.0
Have you had pain during sexual intercourse or other sexual activity?	4	0.0	25.0	50.0
Was sexual activity enjoyable for you?	5	100.0	100.0	100.0

*The number of patients with any grade of symtoms (mild, moderate, and severe) presented in percent (%)

**All cases were matched

*Cervical brachytherapy was given to the primary cervical cancer patients. Vaginal brachytherapy was given to the primary uterine patients

The sexual activity for the 26 (matched) patients with primary uterine tumors with RT was 30.8% at RT start, 42.3% at 3 months after RT and 42.3% 12 months after RT (Table 4). The number of patients who felt less attractive and less feminine due to the treatment or disease was 38.5% 12 months after RT. Few patients answered the questionnaires regarding sexual activity (*N* = 4–5, matched cases) and out of these patients, 75% had symptoms of dry vagina 3 months after RT. Sixty percent had a feeling that the vagina was too tight and 50% felt pain during sexual intercourse 12 months after RT (Table 4).

### Number of patients with symptoms in relation to type of RT treatment

**Fig. 2:**
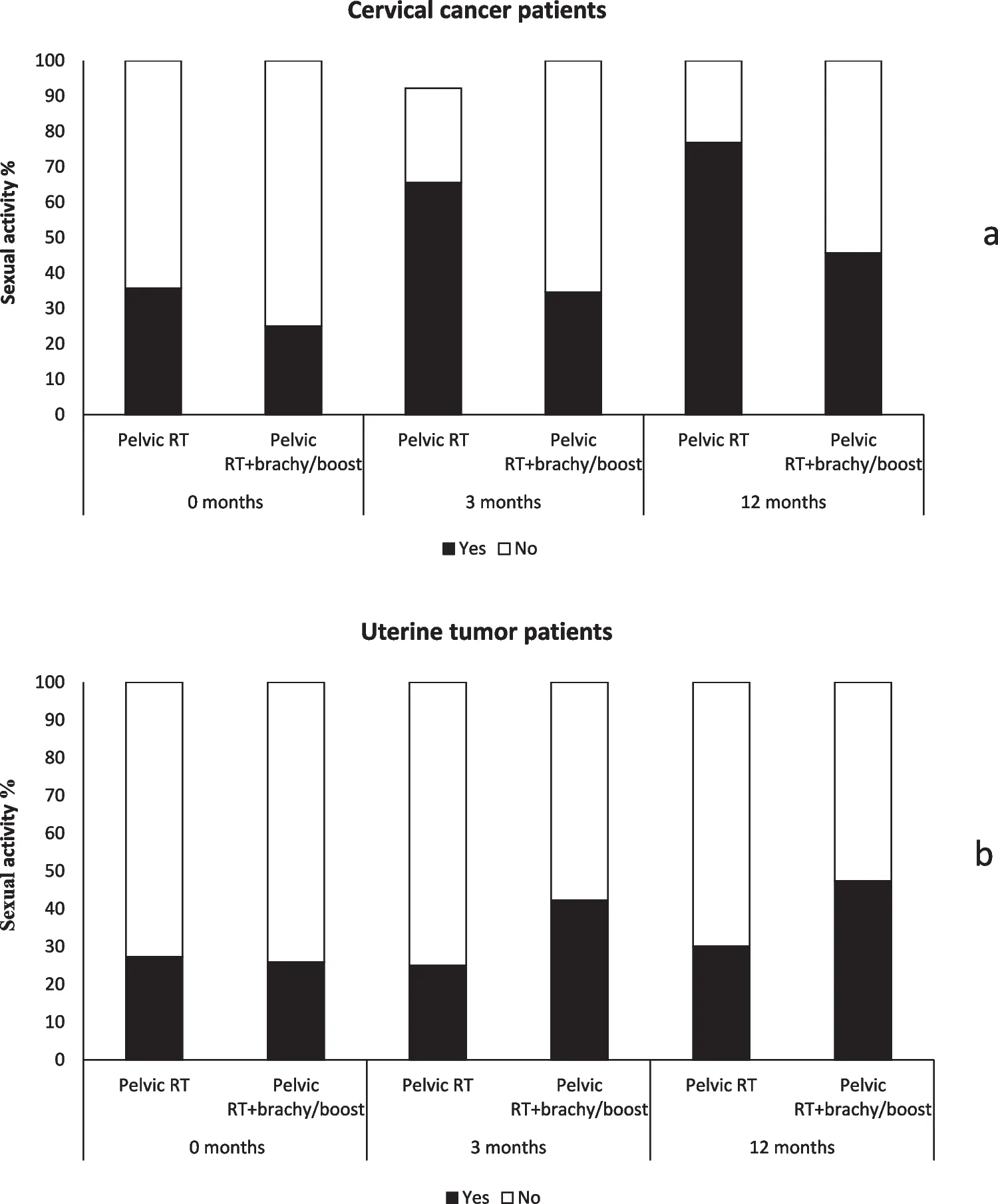
The sexual activity (%) in primary cervical cancer patients (**a**) and primary uterine tumor patients (**b**) with RT alone compared to RT + brachytherapy/boost at RT start, 3 months and 12 months after RT

The patients with sexual symptoms were further studied in relation to type of pelvic RT treatment. In patients with primary cervical cancer, the sexual activity was significantly higher in patients with only pelvic RT compared with those receiving pelvic RT + cervical brachytherapy (CBT)/boost at 3 months (*P* = 0.007, 34.6% vs. 73.3%) and at 12 months after RT (*P* = 0.054, 45.7% vs. 76.9%, Fig. 2). In the group of patients with primary uterine tumors, there was no such significant difference between those with additional vaginal brachytherapy/boost or those who received pelvic RT alone at the start of RT and at 3 and 12 months after RT (*P* > 0.05, Table 5).

**Table 5:** Sexual symptoms in relation to type of RT treatment before RT and at 3 and 12 months after RT by using the ENGOT CX-24 and the ENGOT EN-24 form

Symptoms and type of RT treatment	Time		RT	RT + brachytherapy/boost*	*p*-value
			***n*** (%)	***n*** (%)	
Sexual symptoms ENGOT CX-24					
During the latest 4 weeks, have you been sexually active?	0 months	Yes	5 (35.7)	12 (25.0)	0.429
		No	9 (64.3)	36 (75.0)	
	3 months	Yes	11 (73.3)	19 (34.6)	0.007
		No	4 (26.7)	36 (65.4)	
	12 months	Yes	10 (76.9)	16 (45.7)	0.054
		No	3 (23.1)	19 (54.3)	
Sexual symptoms ENGOT EN-24					
During the latest 4 weeks, have you been sexually active?	0 months	Yes	3 (27.3)	7 (25.9)	0.931
		No	8 (72.7)	20 (74.1)	
	3 months	Yes	3 (25.0)	11 (42.3)	0.304
		No	9 (75.0)	15 (57.7)	
	12 months	Yes	3 (30.0)	9 (47.4)	0.367
		No	7 (70.0)	10 (52.6)	

### Local and systemic estrogen treatment in relation to sexual activity

The local and systemic estrogen treatment was further evaluated in relation to sexual symptoms in primary cervical and uterine tumor patients with RT. Cervical cancer patients who received systemic estrogen treatment due to post-menopausal symptoms had a significantly higher sexual activity before RT, and the effect persisted at 3 and 12 months after RT compared to patients without systemic estrogen treatment (*P* < 0.001, *P* = 0.017, *P* = 0.002). At RT start, cervical cancer patients with local estrogen treatment tended to have a higher frequency of sexual activity compared to patients without local estrogen treatment (*P* = 0.079). No significant relationship was found at 3 months and 12 months after RT (*P* > 0.05). In primary uterine tumors, there were no significant association between sexual activity and systemic or local estrogen treatment at RT start, 3 months, or 12 months after RT (*P* > 0.05).

### Patients functioning

Further the global health status and patients functioning of primary cervical cancer and uterine tumors with RT was evaluated by using the EORTC QLQ-C30 form. In the cervical cancer patients, the global health status, cognitive and emotional functioning were significantly improved 12 months after RT compared to RT start (*P* = 0.008, *P* = 0.045, *p* < 0.001, Table 3). For the uterine tumor patients, the emotional functioning tended to improve at 3 and 12 months after RT (*P* = 0.014, *P* = 0.018, Table 3).

## Discussion

High doses of pelvic RT cause tissue injury as inflammation and fibrosis who leads to problems in vaginal function such as dryness, shortening and tightening of the vagina which could cause discomfort and affect sexual enjoyment and patient’s overall wellbeing. In this longitudinal prospective study, the association between pelvic RT and sexual function was studied in both patients with primary cervical cancer and in patients with primary uterine tumors.

At first, the relationship between sexual activity and different types of RT treatments was studied in the primary cervical cancer patients. Here, we showed that the sexual activity was significantly higher in patients with pelvic RT alone compared to patients with pelvic RT + cervical brachytherapy/boost. The relationship between pelvic RT and side effects as sexual problems has been studied by Kirchheiner et al. (2022). However, in this study, all patients received additional brachytherapy (100%) and no comparisons between RT alone and RT + brachytherapy were therefore performed [11]. As far as we know, this is the first prospective study that shows a significant relationship between sexual activity and type of pelvic RT treatment in primary cervical cancer patients. This difference in sexual activity between the cervical cancer patients with RT + brachytherapy vs. RT alone could partly be attributed to increased development of fibrosis in the vagina because of a higher dose of radiation when the patients are treated with RT + brachytherapy/boost compared to RT alone. The high doses of brachytherapy/boost in addition to RT could also damage the vaginal epithelium and reduce the number of estrogen receptors which further reduces the ability for vaginal lubrication [7]. We suggest that additional cervical brachytherapy/boost has a negative impact on sexual activity in primary cervical cancer patients. Additional brachytherapy/boost gives a higher total radiation dose which increases the risk of developing fibrosis/atrophy of the vaginal epithelium which leads to tightening and dryness of the vagina with further impaired sexual function. However, a larger randomized study is needed to further elucidate this issue.

We further studied the sexual activity in primary uterine tumor patients in relation to type of RT by using the EORTC-QLQ-EN24 form. We found no significant relationship in sexual activity between primary uterine tumor patients with pelvic RT alone and pelvic RT and additional vaginal brachytherapy/boost. Similar findings were reported by Karabuga et al. (2015) who studied 144 primary endometrial cancer patients by using the quality-of-life questionnaire EORTC-QLQ-CX24 [9]. A large, randomized study of primary endometrial cancer patients did not make the comparison between patients with pelvic RT alone and patients with pelvic RT + vaginal brachytherapy/boost in relation to sexual symptoms [12].

There is thus a discrepancy between cervical cancer patients and the patients with primary uterine tumors with regards to the effect of RT on sexual activity. One explanation could be thatthe cervical cancer patients receive a much higher total radiation dose compared to the uterine tumor patients, increasing the risk of development of fibrosis in the vagina. Since, most cervical cancer patients and very few uterine tumor patients are premenopausal, the RT induced reduction in the estrogen production in premenopausal women (which further leads to reduced stimulation/lubrication of the vaginal epithelium) could also in part account for this discrepancy.

Further, we studied the sexual activity in primary cervical cancer who had received curative RT. Here, we showed that the sexual activity increased from RT start to 12 months after RT. Even though the sexual activity increased from RT start to 12 months after RT the frequency of sexual activity was still low (52.5%) 12 months after RT, which is similar results found by others [11]. Studies on an age-matched norm population reported that the sexual activity was 72% which is a high frequency compared to the cervical cancer patients with RT [7]. Since these patients are women in mid-life with a mean age around 50 years, we suggest that the frequency of sexual activity in primary cervical cancer patients is affected by RT and is still at a low level 12 months after RT.

Further, we studied the sexual behavior and perception in cervical cancer patients with RT who were sexually active. Most patients (81.8%) felt that their vagina was dry, 60% felt that their vagina was short, 50% had pain during intercourse and 30% experienced tightening of their vagina 12 months after RT. Similar findings have been reported by others, but with a higher frequency of tightening of the vagina (50% compared to 30% in our study) [11]. Additional cervical brachytherapy was given to all patients (100%) in the previous study compared to only 83.7% in our study. The resulting increased fibrosis could account for the increased tightening of the vagina in the previous study.

At 3 months after RT, 73.7% of the cervical cancer patients worried that sex would be painful. A high frequency of patients 63.4% still felt dissatisfied with their body at 12 months after RT, showing that treatment with high doses of pelvic RT for these patients also affects the psychological aspects of the sexuality with a high frequency of patients experiencing changes in their body image.

The relationship between pelvic RT and sexual function was also studied in patients with primary uterine tumors. Here we showed that the sexual interest increased, and the sexual activity tended to increase 12 months after RT compared to at RT start. However, the frequency of sexual interest and the sexual activity was still at a low level of 40.7% and 42.3% 12 months after RT. These results are in line with a study on endometrial cancer patients with pelvic RT or brachytherapy, where the sexual activity and sexual interest was 39% 12 months after RT [10]. The sexual activity in our study and the previous study was significantly reduced compared to the age-matched norm population for both the Pelvic RT and brachytherapy/boost group [10]. We can thus conclude that although the sexual interest and sexual activity in primary uterine tumors seemed to increase from RT start to 12 months after RT, they remain at a low level 12 months after RT.

Very few matched cases were present in the group of primary uterine tumor patients that were sexually active. Out of these patients, 50% felt that their vagina was dry, 50% reported pain during sexual intercourse and 60% felt that their vagina was too short/tight. In previous reports no detailed information regarding these specific sexual symptoms was presented [4, 12]. However, it is difficult to draw valid conclusions out of our results since there were very few patients with primary uterine tumors who were sexually active and larger prospective studies are recommended to confirm our initial findings.

Next, the use of systemic and local estrogen treatment in relation to sexual activity was studied in primary cervical cancer and uterine tumor patients with RT. Our findings show that treatment of primary cervical cancer patients with systemic estrogens significantly improved the sexual activity before RT, at 3 months and 12 months after RT compared to patients without systemic estrogen treatment [11]. At RT start, cervical cancer patients with local estrogen treatment tended to have an increased sexual activity compared to patients without local estrogen treatment but this increase was not statistically significant. No significant difference was seen either at 3 or 12 months after RT, suggesting that the dose of local estrogen applicated was not sufficient to improve the sexual activity in cervical cancer patients with RT. It was shown in a previous study, that RT reduced the number of estrogen receptors in the vaginal epithelium in cervical cancer patients [17], suggesting that the cervical cancer patients with RT probably need a higher dose of local estrogen in order to get a sufficient stimulation/lubrication of the vaginal epithelium which could lead to improved sexual activity. In the primary uterine patients with RT, there were no significant differences in sexual activity between patients with or without systemic (few cases) or local estrogen treatment. It could be speculated that a higher dose of local estrogens could be of benefit for primary uterine tumor patients with RT. However, further preclinical, and larger randomized clinical studies are needed to clarify this issue.

Taken together, our study suggests cervical cancer patients and primary uterine tumor patients require early medical interventions and attention from the health care. Future care should include treatment with both systemic and local estrogens, local lubricants, usage of vaginal dilatators and sexual advice by curator. Such interventions are even more important for the cervical cancer patients who receive additional cervical brachytherapy/boost.

The strength of this study is that it is prospective and longitudinal, and all patients received the same type of RT treatments with IMRT technique. However, the small sample size, especially in the primary uterine tumor group is a limiting factor.

In conclusion, the sexual activity was significantly higher in primary cervical cancer patients with pelvic RT alone compared to patients with pelvic RT and additional cervical brachytherapy/boost, suggesting that additional cervical brachytherapy/boost has a negative impact on sexual activity in primary cervical cancer patients. No such relationship was seen in the primary uterine tumors. Very few of the cervical cancer patients 52.5%, and 43.2% of the uterine tumor patients were sexually active 12 months after RT. Treatment of primary cervical cancer patients with systemic estrogens significantly improved the sexual activity before RT, at 3 months and 12 months after RT compared to patients without systemic estrogen treatment. However, treatment with local estrogens did not improve the sexual activity in the cervical cancer or in uterine tumor patients after RT. Future care should include use of systemic and local estrogens, local lubricants, vaginal dilatators, and sexual advice by curator, especially for the cervical cancer patients who receive brachytherapy/boost.

## Data Availability

Data is provided within the manuscript or supplementary information files.
